# A new 3D myeloarchitectonic map of the human neocortex based on data from the Vogt–Vogt school

**DOI:** 10.1007/s00429-023-02671-6

**Published:** 2023-06-28

**Authors:** Rudolf Nieuwenhuys, Cees A. J. Broere

**Affiliations:** 1 The Netherlands Institute for Neuroscience, Royal Netherlands Academy of Arts and Sciences, Meibergdreef 47, 1105 BA Amsterdam, The Netherlands; 2 Spinoza Centre for Neuroimaging, Meibergdreef 75, 1105 BK Amsterdam, The Netherlands; 3 Department of Medical Imaging, Anatomy, Radboud University Medical Center, Postbus 9101, 6500 HB Nijmegen, The Netherlands

**Keywords:** Architectonics, Cytoarchitectonics, Myeloarchitectonics, Neocortex, Neuroimaging, Weigert technique

## Abstract

During the period extending from 1900 to 1970, Oskar and Cécile Vogt and their numerous collaborators (‘the Vogt–Vogt school’) published a large number of studies on the myeloarchitecture of the human cerebral cortex. During the last decade, we have concerned ourselves with a detailed meta-analysis of these now almost totally forgotten studies, with the aim to bringing them into the modern era of science. This scrutiny yielded *inter alia* a myeloarchitectonic map of the human neocortex, showing a parcellation into 182 areas (Nieuwenhuys et al. in Brain Struct Funct 220:2551–2573, 2015; Erratum in Brain Struct Funct 220: 3753–3755, 2015). This map, termed 2D’15, which is based on data derived from all of the 20 publications constituting the myeloarchitectonic legacy of the Vogt–Vogt school, has the limitation that it is two-dimensional i.e. it shows only the parts of the cortex exposed at the free surface of the cerebral hemispheres and not the extensive stretches of cortex hidden in the cortical sulci. However, a limited set of data, derived from four of the 20 publications available, has enabled us to create a 3D map, showing the myeloarchitectonic parcellation of the entire human neocortex. This map, designated as 3D′23, contains 182 areas: 64 frontal, 30 parietal, 6 insular, 19 occipital and 63 temporal. We have also prepared a 2D version (2D′23), of this 3D′23 map to serve as a link between the latter and our original 2D′15 map. Detailed comparison of the parcellations visualized in our three maps (2D′15, 2D′23 and 3D′23) warrants the conclusion that our new 3D′23 map may be considered as representative for the entire myeloarchitectural legacy of the Vogt–Vogt School. Hence it is now possible to compare the rich amount of myeloarchitectonic data assembled by that school directly with the results of current 3D analyses of the architecture of the human cortex, such as the meticulous quantitative cyto- and receptor architectonic studies of Zilles, Amunts and their numerous associates (Amunts et al. in Science 369:988–992, 2020), and the multimodal parcellation of the human cortex based on magnetic resonance images from the Human Connectome Project, performed by Glasser et al. in Nature 536:171–178, 2016).

**Supplementary Information:**

The online version contains supplementary material available at 10.1007/s00429-023-02671-6.

## Introduction

During the period extending from 1900 to 1970, the German–French research couple Oskar and Cécile Vogt and their numerous collaborators (‘The Vogt–Vogt school’) worked on a comprehensive research programme aimed at the parcellation of the mammalian cerebral cortex into fundamental morphological and potentially functional units. Their approach included systematic studies on the local differences in the size, shape and distribution of the cortical cells, using the Nissl stain (cytoarchitectonics), with corresponding studies based on Weigert-stained material, on the local variations of the patterns formed by the cortical myelinated fibres (myeloarchitectonics).

The study of the cytoarchitecture of the cortex was delegated to Korbinian Brodmann, who joined the Vogts in 1901 and remained attached to their laboratory until 1909. Brodmann published an impressive series of studies on the cytoarchitecture of the cortex of a considerable number of mammals, including the hedgehog, lemur, guenon and man, and a summarizing monograph (Brodmann 1909), containing his famous map of the human cortex, showing a parcellation into 43 cytoarchitectonic areas, which were designated with Arabic numerals.

The myeloarchitectonic part of the research programme was initiated by Oskar Vogt himself. Focusing on the human brain, he analyzed the frontal (Vogt 1910), parietal (Vogt 1911) and insular lobes (Vogt and Vogt 1911), in which he identified 66, 30 and 6 myeloarchitectonic areas, respectively. His work was continued by numerous collaborators, all of whom confined themselves to the study of the myeloarchitecture of a single lobe of the human telencephalic hemisphere. For a complete list of these authors and their 20 publications we refer to Table 1 in Nieuwenhuys et al. (2015a); here we confine ourselves to four representative examples: Strasburger (1937, frontal lobe, 64 areas), Gerhardt (1940, parietal lobe, 30 areas), Lungwitz (1937, occipital lobe, 17 areas) and Hopf (1954, temporal lobe, 62 areas). From the results of these studies, Vogt and Vogt concluded that the total number of areas or topistic units in the human cortex amounts to about 200 (Vogt and Vogt 1919; Vogt 1951). They were deeply convinced that cytoarchitectonics and myeloarchitectonics represent complementary approaches which lead categorically to identical results (Vogt and Vogt 1919, 1954, 1956). The discrepancy between the relatively low number of cytoarchitectonic areas in the human cortex distinguished by Brodmann (1909), and the much higher number resulting from their own myeloarchitectonic studies, was explained by claiming that Brodmann had overlooked many areal boundaries (Vogt 1918). Vogt and Vogt (1919) emphasized the finding of cytoarchitectonic counterparts of all of their myeloarchitectonic areas. It is relevant in this context that three of their collaborators, Gerhardt (1940), Brockhaus (1940) and Sanides (1962), who carried out combined cytoarchitectonic and myeloarchitectonic analyses of the human frontal, insular and parietal cortices, respectively, all reported a complete concordance of the results obtained with the two approaches.

**Table 1:** The myeloarchitectonic studies of the human neocortex on which the construction of our 3D′23 map is based

Author	Structure	Brain analyzed	Number of sections presented
Strasburger (1937)	Frontal lobe	A 39 R	29
Batsch (1956)	Parietal lobe	A 37 L	18
Lungwitz (1937)	Occipital lobe	A 37	21
Hopf (1954)	Temporal lobe	B 59	18

A, brains belonging to the Vogt–Vogt collection; B, brain belonging to the collection of K. Kleist; R, right hemisphere; L, left hemisphere

The results of the cytoarchitectonic- and myeloarchitectonic parts of the research programme of the Vogt–Vogt school have been separately judged and, as we shall see, highly differentially appreciated by the neuroscience community. Brodmann’s (1909, 1914) map in which the human cortex, as already mentioned, was partitioned into 43 cytoarchitectonic areas, became world famous and represents up to the present by far the most widely used reference system for the localization of cortical functions. The results of the myeloarchitectonic studies of the Vogt–Vogt school, on the other hand, were met with great mistrust and skepticism. Thus, Bailey and Von Bonin (1951), who attempted to realize a combined cyto- and myeloarchitectonic analysis of the human cortex, admitted to have failed to obtain reliable results with the Weigert technique, and declared that, so far as the architectonics of the cortex is concerned, only the Nissl technique yields constant results. Le Gros Clark (1952, p. 104) stated that “the incredibly complicated maps of cortical areas elaborated by the Vogt school should be regarded with the greatest suspicion”, and Sholl (1956, p. 24) declared that “C. and O. Vogt and their pupils carried their process of subdivision to an extreme that can only be described as fantastic”. It was generally felt that Brodmann’s subdivision came somewhere near to what is’reasonable’, and that the much more detailed myeloarchitectonic parcellations reported by the Vogts represent the fruits of a strange, esoteric subculture rather than the results of sound scientific research. Hence, the Brodmann cytoarchitectonic map became an icon of neuroscience, whereas the results of the myeloarchitectonic analyses of the cortex sank into oblivion.

We are convinced that the criticism leveled in the literature against the myeloarchitectonic cortex studies of the Vogts and their associates is fully undeserved and is due to (1) lack of acquaintance with the quirks of the Weigert technique, (2) lack of experience with the myeloarchitectonic approach and (3) difficulties with the extremely complex (German !) myeloarchitectonic vocabulary encompassing more than 80 terms (Wahren 1960). There can be no doubt that myeloarchitectonics, as practised by the Vogt–Vogt school, represents an absolutely sound neuroanatomical subdiscipline. Hence, the present authors started about ten years ago a detailed meta-analysis of the now almost totally forgotten myeloarchitectonic cortex studies of the Vogts, with the intention to render them accessible to current research, which pursues a goal comparable to that of the Vogts, viz. the creation of a unified map of the architecture of the human neocortex. This scrutiny has yielded the following results so far: (1) A compilation and re-evaluation of the myeloarchitectonic data available (Nieuwenhuys 2013). (2) A new myeloarchitectonic map of the human neocortex, designated as 2D′15 (Nieuwenhuys et al. 2015a, b). (3) A map, termed 2D′17, showing the overall myelin content of the individual architectonic areas in the human neocortex (Nieuwenhuys and Broere 2017), and (4) a detailed comparison of the cytoarchitectonic and myeloarchitectonic maps of the human cortex produced by the Vogt–Vogt school, resulting in the composition of a combined cyto-myeloarchitectonic map (Nieuwenhuys and Broere 2020: 2D′20).

All of the three maps produced by us so far have in common that they show only the parts of the cortex exposed at the free surface of the cerebral hemispheres, a feature which they share with all classical maps of the neocortex, including those of Brodmann (1909), Von Economo and Koskinas (1925), Bailey and Von Bonin (1951) and Sarkissov et al. (1955). This is an important limitation indeed, because a considerable part of the cortex is known to border on the surface hidden in the cortical sulci (Zilles et al. 1988). However, the legacy of the Vogt–Vogt school appeared to include a few papers containing data rendering it possible to produce a three-dimensional myeloarchitectonic map, termed 3D′23, encompassing the entire, i.e. the hidden as well as the superficially situated parts of the cortex. The present paper is devoted to the production, presentation and validation of this new 3D map.

## Material and methods

### Material

**Fig. 1:**
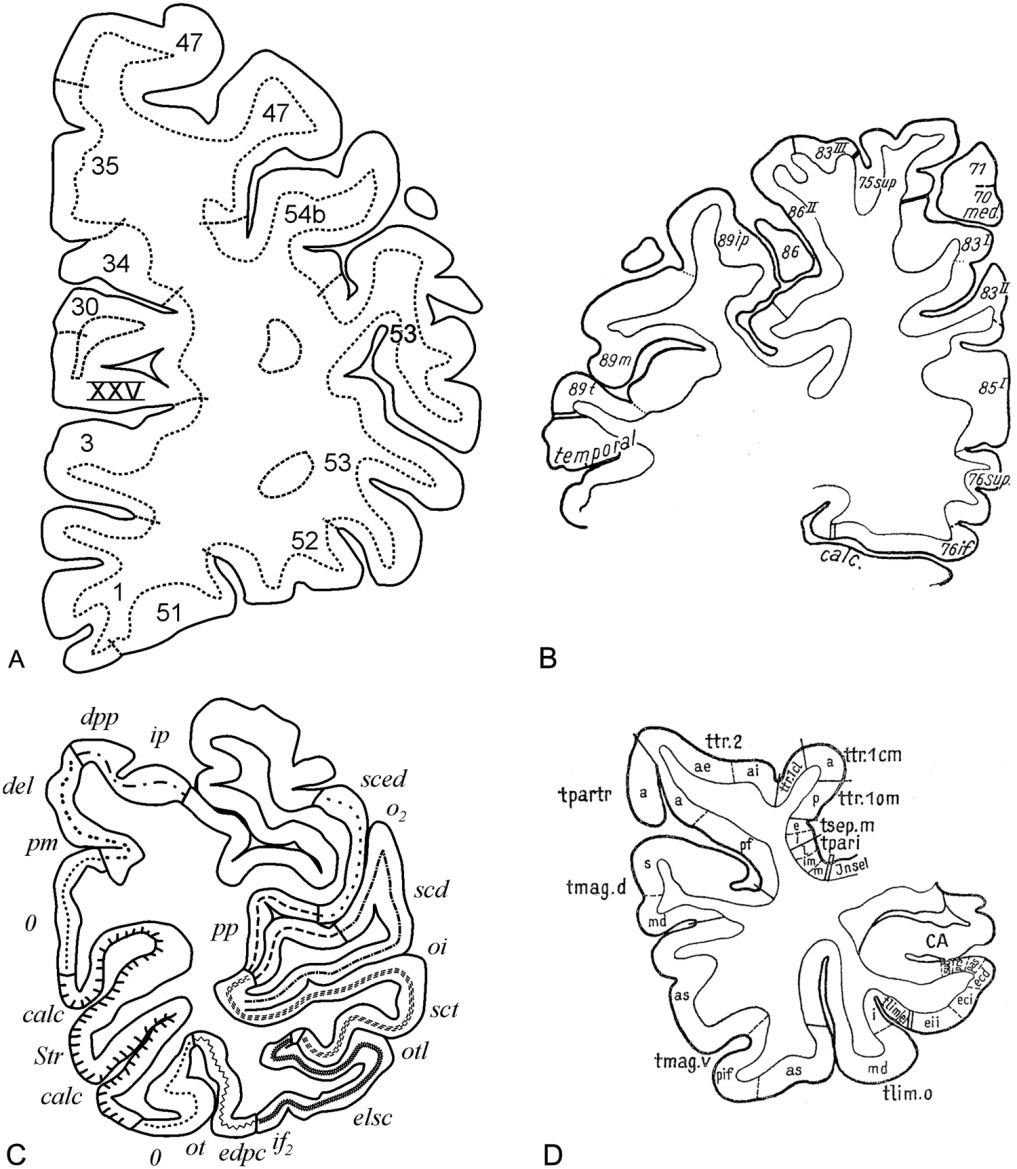
Representative diagrammatic transverse sections through the various cerebral lobes in which the myeloarchitectonic parcellation is indicated. **A** Frontal lobe (Strasburger 1937); **B** parietal lobe (Batsch 1956); **C** occipital lobe (Lungwitz 1937); **D** temporal lobe (Hopf 1954)

The total myeloarchitectonic legacy of the Vogt–Vogt school encompasses 20 publications, which are listed in Table 1 in Nieuwenhuys et al. (2015a). All of these publications relate to the cortex of one of the cerebral lobes and all contain maps showing the various aspects of the lobe studied, for instance the lateral, superior, medial and basal aspects of the frontal lobe. It is on these so-called lobar aspect maps that our 2D′15 map was based. However, four of the 20 publications available, listed here in Table 1, contain, apart from lobar aspect maps, sets of diagrammatic drawings, derived from Weigert preparations, in which the results of the pertinent myeloarchitectonic analyses are indicated (Fig. 1). It is these sets of labeled diagrammatic sections which enabled us to create our new 3D′23 myeloarchitectonic map of the entire human neocortex.

### Introduction of a standard reference brain

Because the data to be employed are derived from three different brains (see Table 1), we introduced a standard reference brain to be used as a template to which all data are to be transferred. As such, we have selected one of the brain templates provided by the Montreal Neurological Institute, viz*.* Colin27, which is usually referred to as the ‘single subject MNI template’. This template has also been used during the preparation of our 2D′15 map. For an atlas of this Colin27 brain, including standard lateral, superior, medial and basal views, we refer to a previous publication (Nieuwenhuys et al. 2015a, Figs. 2, 3, 4, 5, 6). Images of 187 equidistant frontal sections through this brain, designated from now onward as ‘Colin sections’, are available in the literature (Evans et al. 1993).

**Fig. 2:**
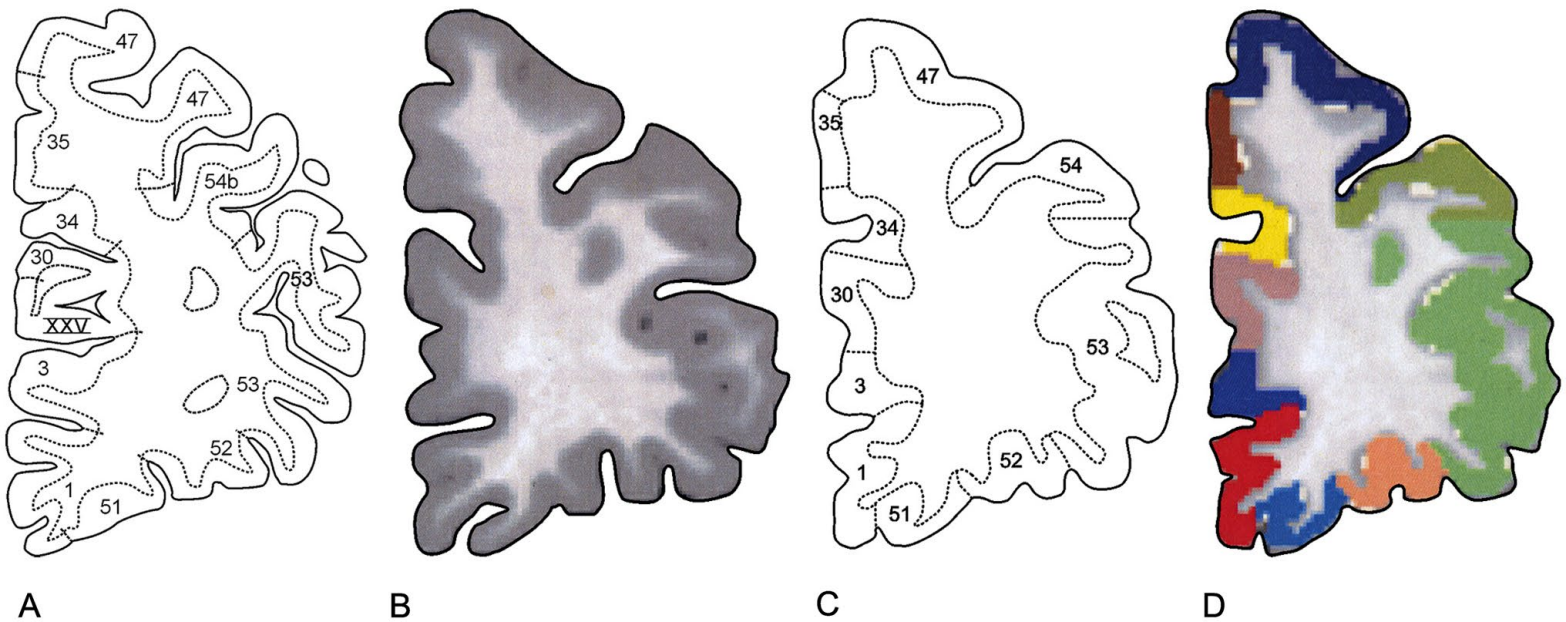
The transfer of the myeloarchitectonic parcellation of a section through the frontal lobe, as presented by Strasburger (1937), to a corresponding section of the Colin brain. **A** Section of the Strasburger brain representing the mirror image of the section shown in Fig. 1A; **B** corresponding section of the Colin brain; **C** tracing of the section shown in B to which the parcellation shown in **A** is transferred; **D** section through the Colin brain in which the parcellation indicated with numbers in **C** is ‘translated’ into colors

**Fig. 3:**
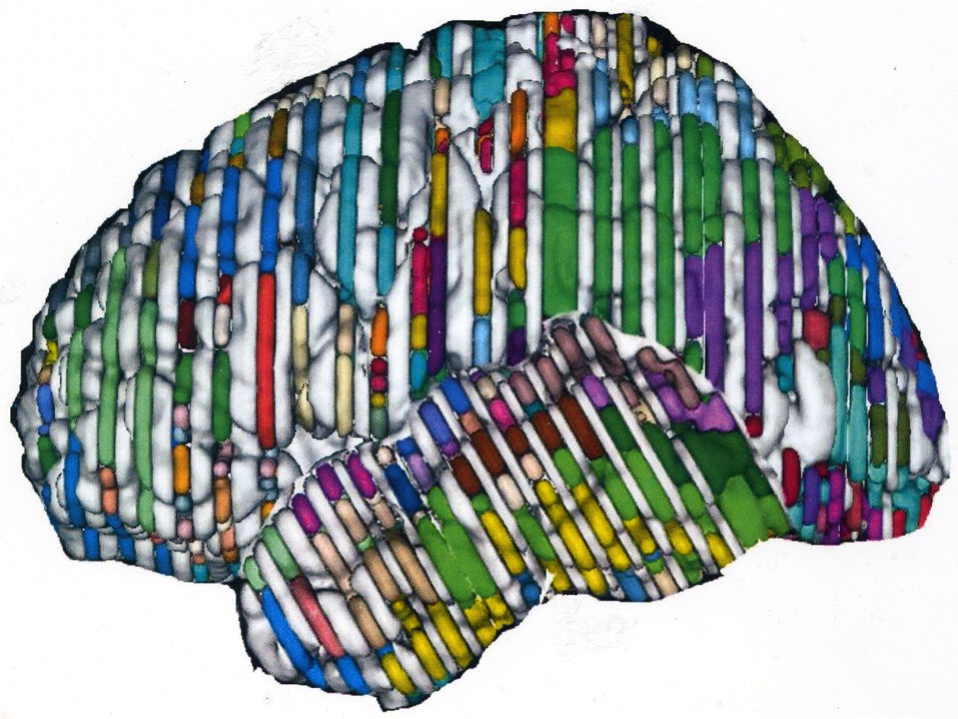
Lateral view of the Colin brain in which the color-coded sections of the various lobes are placed in their appropriate positions

**Fig. 4:**
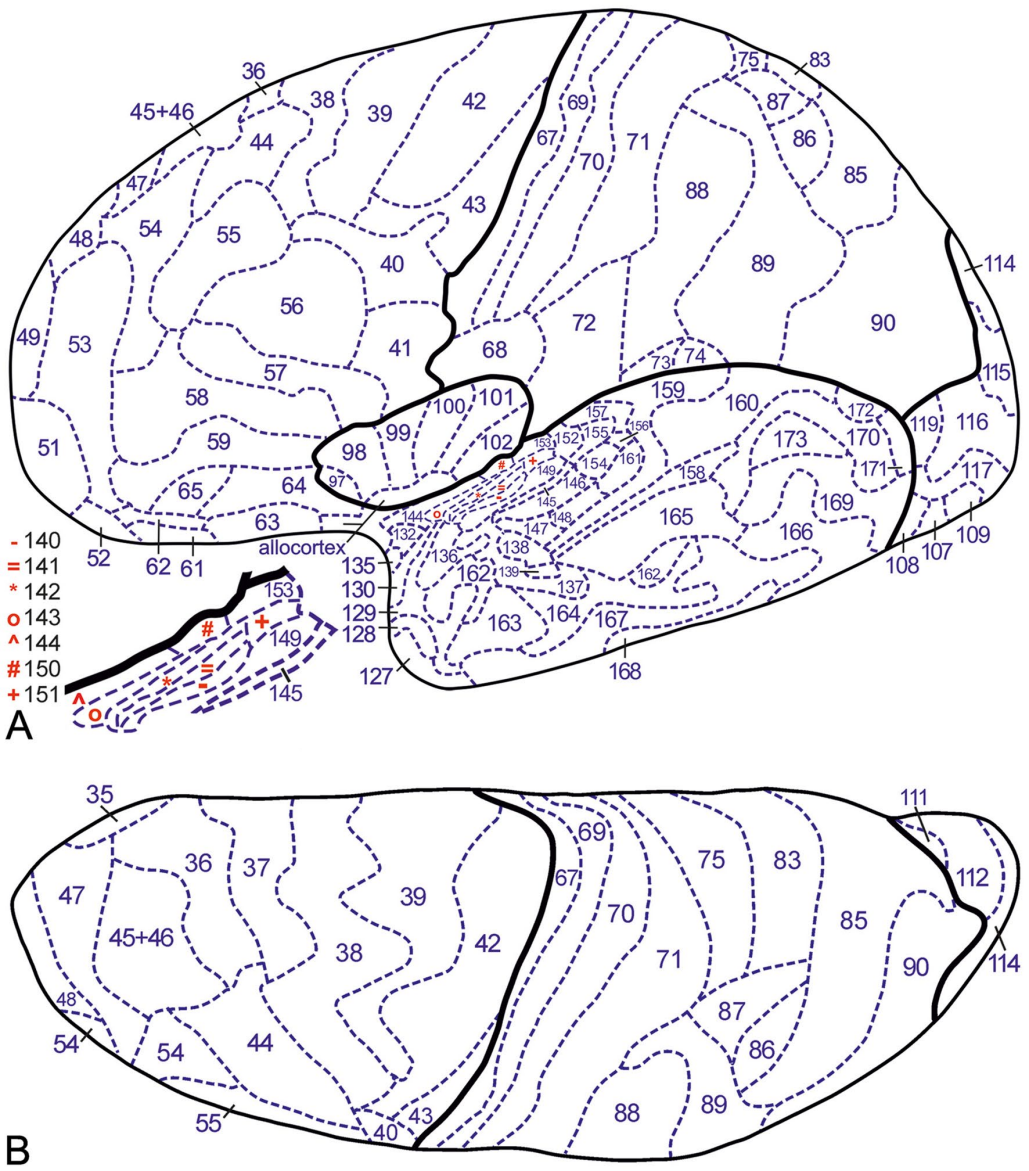

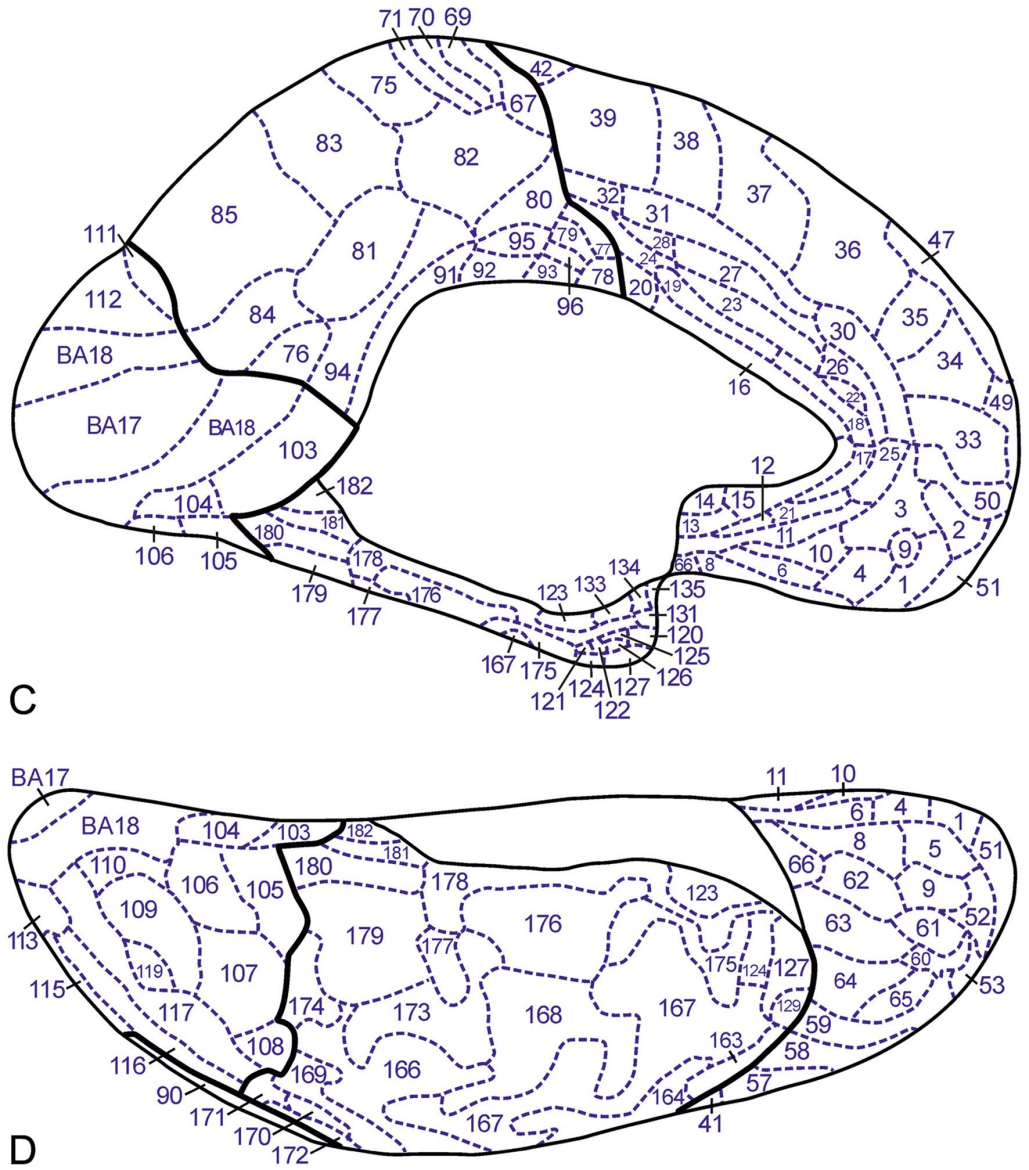
Inflated lateral (**A**) and superior (**B**) views of our D3′23 map. Inflated medial (**C**) and basal (**D**) views of our D3′23 map

**Fig. 5:**
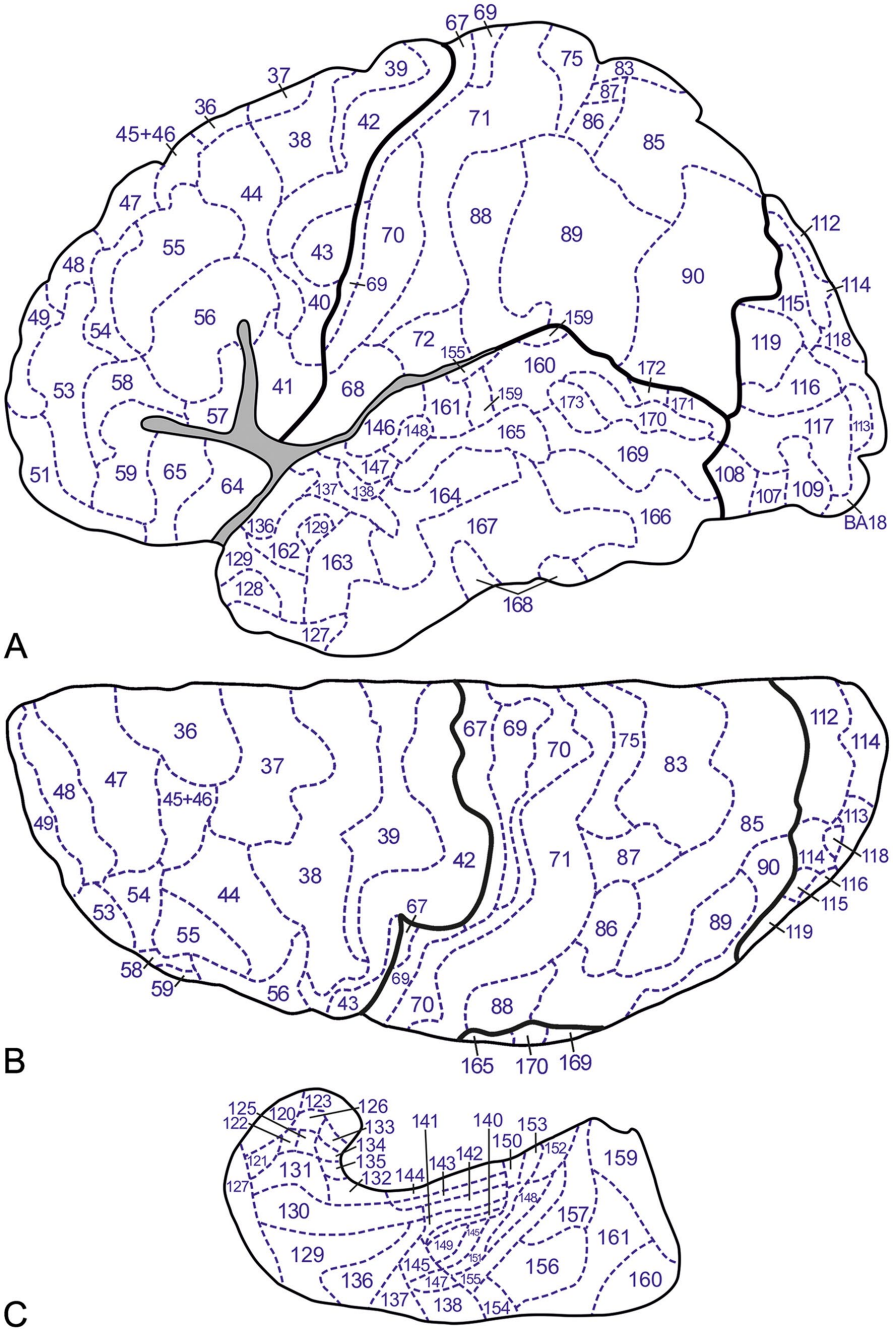

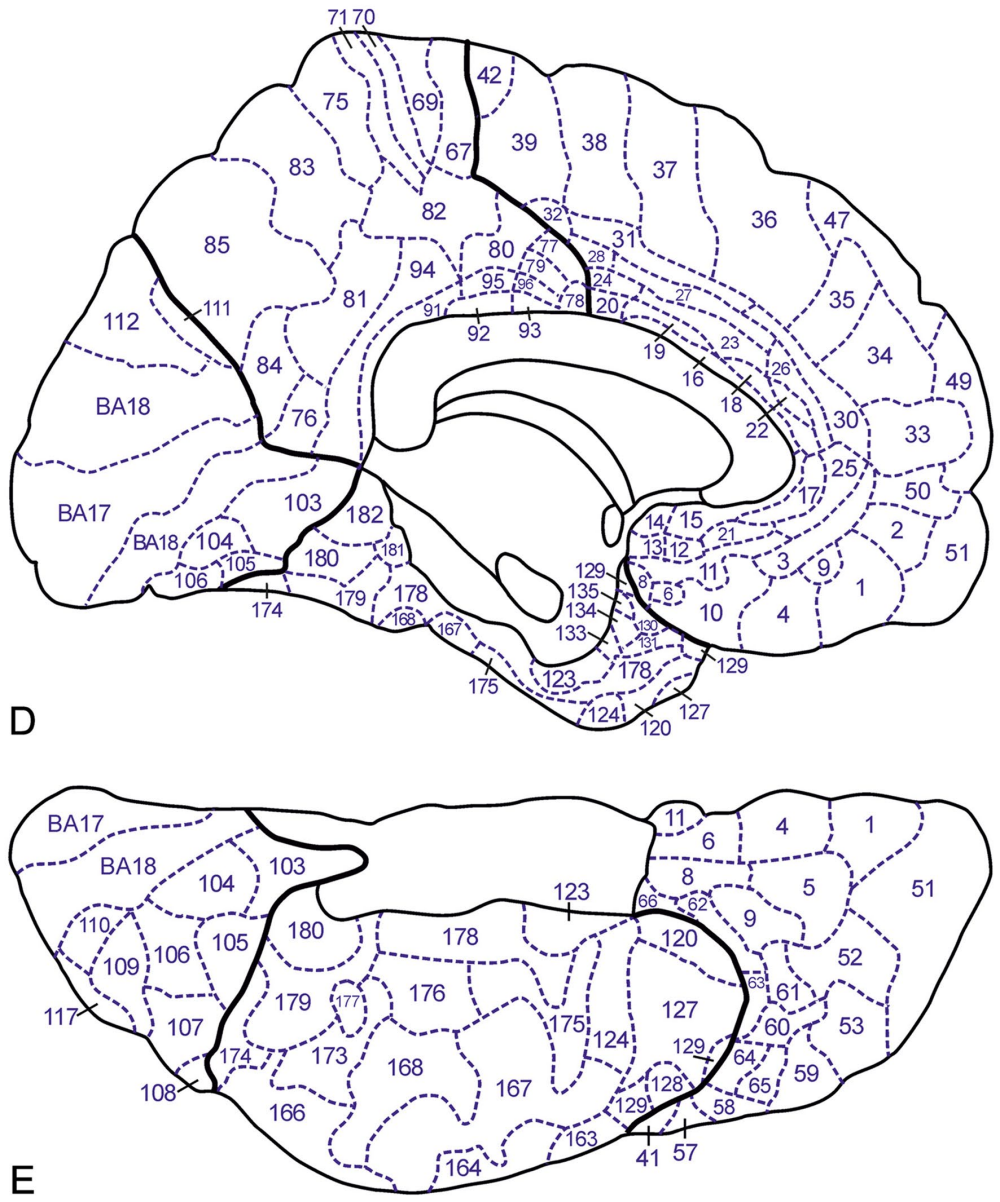
Lateral view (**A**), superior view (**B**) and superior view of the planum temporale (**C**) of our 2D′23 map. Medial (**D**) and basal (**E**) views of our 2D′23 map

### Matching of the diagrammatic, labeled sections (DLSs) with the Colin sections (CSs)

Reference to Table 1 shows that 29 DLSs through the frontal lobe, 18 through the parietal lobe, and 21 through the occipital lobe were available. We succeeded in finding an acceptable CS match for each of these DLSs, based on anteroposterior positional- and configurational correspondence.

The direction of sectioning of the 18 DLSs through the temporal lobe available, appeared to make an angle of 32 degrees with that of the anteroposterior CSs. We adapted the direction of sectioning of the latter, i.e. the Colin27 temporal lobe, to that of the temporal DLSs using ITK-SNAP version 3.6.0 software (Yushkevich et al. 2006), and succeeded in finding a suitable ‘adapted’ CS match for each of the 18 temporal DLSs.

### Transfer of the myeloarchitectonic information incorporated in the DLSs to their CSs equivalents

The DLSs and their corresponding CSs were placed side by side (Fig. 2A, B), and the myeloarchitectonic parcellation indicated in the former, including their respective number or letter codes, was transferred to the latter using ITK-SNAP software (Fig. 2C). Thus, the respective Colin sections were changed into *labeled* Colin sections (LCSs). Next, the number- or letter codes of the various areas were replaced by specific colors (Fig. 2D). Finally, a thickness of a few millimetres was attributed to each of the LCSs, and the coding colors of the various myeloarchitectonic areas were extended over the lateral surfaces of the three-dimensionalized sections. It may be added that the cortical foldings did not play an active role in the transfer of the myeloarchitectonic areas. However, if in a particular DLs the boundary of a myeloarchitectonic area appeared to correspond with the bottom of a cortical folding, and if the corresponding cortical region in the corresponding CS contained a topologically corresponding cortical folding, then the pertinent correspondence was maintained in that CS.

### Preparation of a 3D myeloarchitectonic map (3D′23)

Using the ITK-SNAP program, a 3D representation from the Colin27 template, labeled as indicated above, a mesh file was exported, containing the complete surface of the Colin27 hemisphere with the markings indicating the exact locations of the cortical areas. This mesh file was transferred, using Cosmetic vl.15.cbs (Yang et al. 2012) to a non-inflated and an inflated Colin27 surface, including the markings indicating the exact locations of the myeloarchitectonic cortical areas, resulting in 2D and 3D myeloarchitectonic maps (2D′23 and 3D′23). Figure 5 presents the non-inflated brain, Fig. 4 the inflated brain. The origin and application of the number codes used in these figures is explained in paragraph 7.

### Preparation of a 2D myeloarchitectonic map (2D′23)

Standard anatomical projections from the 3D myeloarchitectonic map produced a 2D myeloarchitectonic map (2D′23). Therefore the three-dimensionalized, labeled Colin sections (LCSs), the preparation of which has been described above, were placed in their appropriate antero-posterior sequence and distance, and adjusted to the lateral (Fig. 3), superior, medial and basal views of the Colin27 template, provided in a previous publication (Nieuwenhuys et al. 2015a).

### The coding of the myeloarchitectonic areas

Oskar Vogt was the first investigator who studied the myeloarchitecture of the frontal (Vogt 1910), parietal (Vogt 1911) and insular lobes (Vogt and Vogt 1911). He delineated 66 myeloarchitectonic areas in the frontal lobe, which he designated with the Arabic numbers 1–66, including 30 parietal areas numbered 67–96, and 6 insular areas numbered 97–102. These designations were generally accepted, with the reservation that none of the later students of the myeloarchitecture of the frontal lobe were able to identify Vogts’ areas 7 and 29. For this reason these two areas are lacking in our maps.

As is well known, Brodmann (1909) divided the human occipital cortex into three concentrically arranged cytoarchitectonic areas, the area striata (area 17), the area occipitalis (area 18) and the area praeoccipitalis (area 19). Lungwitz (1937) found that the boundaries between the three occipital cytoarchitectonic areas of Brodmann have distinct myeloarchitectonic counterparts, but he confined his analysis to the praeoccipital area, in which he delineated 17 myeloarchitectonic areas. He designated these areas with combinations of two to four letters, such as pc, del, and elsc. We decided to extend the numbering of myeloarchitectonic areas introduced by Vogt, over the entire neocortex and designated the 17 praeoccipital areas distinguished by Lungwitz with the numbers 103–119 (Nieuwenhuys et al. 2015a, b). For the correspondence between Lungwitz’ designations and our numbering we refer to Table 3 in Nieuwenhuys et al. (2015a). For the two occipital areas not analyzed by Lungwitz, we maintained Brodmann’s numbers: (BA) 17 and 18 (Figs. 4, 5).

Hopf (1954) distinguished 63 myeloarchitectonic areas in the temporal lobe, which he designated with extensive full Latin names and corresponding abbreviations. Continuing the numbering scheme of Vogt (1910, 1911), we indicated these areas with the numbers 120–182 (Nieuwenhuys et al. 2015a, b). For the correspondence of these numbers with Hopf’s abbreviations, we refer to Table 4 in Nieuwenhuys et al. (2015a).

## Results

Here we present two new myeloarchitectonic maps of the human neocortex, a 3D map, termed 3D′23 and a 2D map (2D′23). Both of these maps comprise 182 areas: 64 frontal, 30 parietal, 6 insular, 19 occipital and 63 temporal (Figs. 4, 5). The 3D′23 map, the composition of which has been, as we have seen, rather complex, represents the end of our long quest to prepare an optimal pictorial summary of the myeloarchitectonic studies of the Vogts and their associates, being directly comparable to current 3D parcellations of the human neocortex. However, it must be admitted that this new 3D′23 map is based on a limited dataset (see Table 1), and that hence the question arises whether it is really representative of the rich Vogt–Vogt legacy as a whole. In order to address this question, we prepared the 2D′23 map already mentioned, and involved our 2D′15 map as *tertium comparationum*. The 2D′23 map stands intermediate between the two other maps. It is based on the same dataset as our 3D′23 map, but the results of its myeloarchitectonic parcellation and that of our 2D′15 map are both aligned to the same, i.e. the MNI-Colin27 template. It is important to remember that the 2D′15 map is based on the *entire dataset* of the Vogt–Vogt school. Because we have seen that all of the myeloarchitectonic areas identified are present in all three of the pertinent maps, these findings warrant the conclusion that the myeloarchitectonic parcellation, shown in our 3D′23 map, is representative indeed for the entire body of myeloarchitectonic information assembled by the Vogt–Vogt school.

A remarkable feature of the mapping studies of the four authors on which the present publication is based (see Table 1), is that none of these revealed any area entirely confined to the cortex bordering the lobar sulci. Hence, they added no ‘new’ areas to those already delineated in our 2D studies.

## Discussion

The meta-analysis of the myeloarchitectonic studies on the human cortex of the Vogt–Vogt school, communicated in the present series of publications is, as already mentioned, intended to bring this almost totally forgotten but very valuable body of information into the modern era of science. However, it should be admitted that the starting material available for our meta-analysis imposed several limitations on the outcome of our enterprise. Thus, for the judgment of the myeloarchitecture of the frontal, parietal and insular lobes, two or more publications were available, whereas the material for the analysis of the occipital as well as the temporal lobes was confined to a single study. Moreover, the parcellation of the occipital lobe is confined to the equivalent of Brodmann’s area 19, and finally, the material was too small to address questions concerning the interhemispheric and interindividual variability of the various myeloarchitectonic areas. Nevertheless, we are convinced that our account and its numerous accompanying illustrations represent an adequate survey of the rich myeloarchitectonic legacy of the Vogts and their numerous associates. It may presumably even be qualified as unique, because the chance that any group of investigators will ever repeat this enormous amount of time-intensive and labour-intensive research is nil.

[Note. Foit et al. (2022) claim to have produced a whole-brain 3D myeloarchitectonic atlas of the human cortical surface, based on the Vogt–Vogt legacy. However, this is in our opinion no true 3D map, because the authors had no data on the myeloarchitecture of the large stretches of cortex hidden in the cortical sulci. They actually extrapolated the myeloarchitecture of these hidden parts of the cortex from the adjacent, surface-exposed cortical regions.]

### Perspective

It is our intention to compare the results of the present publication with those of two other recent 3D studies of the parcellation of the human cortex, viz*.* (1) those of Karl Zilles and Katrin Amunts and their numerous associates, which are aimed at producing a probabilistic map of the human neocortex, based on observer-independent, computerized quantitative cyto- and receptor architectonic analyses (Amunts and Zilles 2015; Amunts et al. 2007, 2020; Palomero-Gallagher and Zilles 2018; Zilles and Amunts 2009), and (2) the comprehensive multimodal parcellation of the human cortex, based on magnetic resonance images of 210 healthy young adults participating in the Human Connectome Project, published some years ago by Glasser et al. (2016). It is noteworthy that all three of these groups of investigators arrived at a partition of the human neocortex into about 180 areas.

Hopefully, the results of these comparisons will ultimately contribute to the construction of the unified canonical map, which the Vogts had originally in mind.

### Software

The 2D′23 map is saved as 3DCortex15052023.vtk, the 3D′23 as 3DCortex15052023inf.vtk. The additional GIFTI files are 3DCortex15052023centered.surf.gii, 3DCortex15052023inflated.surf.gii and 3DCortex15052023myeloarchitecture.shape.gii. Cosmetic is found in file cosmetic-release-160530.tar.

### Supplementary Information

Below is the link to the electronic supplementary material.

Supplementary file1 (GII 3430 KB)

Supplementary file2 (GII 3430 KB)

Supplementary file3 (VTK 11512 KB)

Supplementary file4 (GII 2715 KB)

Supplementary file5 (VTK 11511 KB)

Supplementary file6 (GII 59 KB)

Supplementary file7 (ZIP 243390 KB)

## Data Availability

The files 3DCortex15052023.vtk, 3DCortex15052023inf.vtk, 3DCortex15052023centered.surf.gii, 3DCortex15052023inflated.surf.gii, 3DCortex15052023myeloarchitecture.shape.gii, cosmetic-release-160530.tar are available (supplementary material).
