# Supplementary material for: A new 3D myeloarchitectonic map of the human neocortex based on data from the Vogt–Vogt school
Source: Brain Struct Funct. 2023 Jun 28;228(6):1549–59. doi: 10.1007/s00429-023-02671-6 (PMC10751253; doi:10.1007/s00429-023-02671-6)
Supplement: Supplementary file 7 — Supplementary file7 (ZIP 243390 KB) [file 429_2023_2671_MOESM7_ESM.zip › 429_2023_2671_MOESM7_ESM.tar!/cosmetic/jre/Welcome.html]

Welcome to the Java(TM) Platform


## Welcome to the Java^TM^ Platform

Welcome to the Java^TM^ Standard Edition Runtime
Environment. This provides complete runtime support for Java applications.

The runtime environment includes the Java^TM^
Plug-in product which supports the Java environment inside web browsers.

### References

See the Java Plug-in product
documentation for more information on using the Java Plug-in product.

See the Java Platform web site for
more information on the Java Platform.

---


Copyright (c) 2006, 2015, Oracle and/or its affiliates. All rights reserved.
